# Ordered Regions of Channel Nucleoporins Nup62, Nup54, and Nup58 Form Dynamic Complexes in Solution*

**DOI:** 10.1074/jbc.M115.663500

**Published:** 2015-05-29

**Authors:** Alok Sharma, Sozanne R. Solmaz, Günter Blobel, Ivo Melčák

**Affiliations:** From the Laboratory of Cell Biology, Howard Hughes Medical Institute, The Rockefeller University, New York, New York 10065

**Keywords:** biophysics, cell biology, nuclear pore, nuclear transport, protein assembly, FG nucleoporins, Light scattering, Nuclear Pore Complex, Ring cycle hypothesis, Transport channel

## Abstract

Three out of ∼30 nucleoporins, Nup62, Nup54, and Nup58, line the nuclear pore channel. These “channel” nucleoporins each contain an ordered region of ∼150–200 residues, which is predicted to be segmented into 3–4 α-helical regions of ∼40–80 residues. Notably, these segmentations are evolutionarily conserved between uni- and multicellular eukaryotes. Strikingly, the boundaries of these segments match our previously reported mapping and crystal data, which collectively identified two “cognate” segments of Nup54, each interacting with cognate segments, one in Nup58 and the other one in Nup62. Because Nup54 and Nup58 cognate segments form crystallographic hetero- or homo-oligomers, we proposed that these oligomers associate into inter-convertible “mid-plane” rings: a *single large* ring (40–50 nm diameter, consisting of eight hetero-dodecamers) or *three small* rings (10–20 nm diameter, each comprising eight homo-tetramers). Each “ring cycle” would recapitulate “dilation” and “constriction” of the nuclear pore complex's central transport channel. As for the Nup54·Nup62 interactome, it forms a 1:2 triple helix (“finger”), multiples of which project alternately up and down from mid-plane ring(s). Collectively, our previous crystal data suggested a copy number of 128, 64, and 32 for Nup62, Nup54, and Nup58, respectively, that is, a 4:2:1 stoichiometry. Here, we carried out solution analysis utilizing the entire ordered regions of Nup62, Nup54, and Nup58, and demonstrate that they form a dynamic “triple complex” that is heterogeneously formed from our previously characterized Nup54·Nup58 and Nup54·Nup62 interactomes. These data are consistent both with our crystal structure-deduced copy numbers and stoichiometries and also with our ring cycle model for structure and dynamics of the nuclear pore channel.

## Teaser

**Background:** The ordered region (∼150–200 residues) of each channel nucleoporin is subdivided into segments of ∼40–80 residues.

**Results:** In solution, ordered regions associate into dynamic and heterogeneous complexes utilizing previously identified interactions between cognate segments.

**Conclusion:** Solution data are consistent with channel model reconstructed from crystal structures of cognate segments.

**Significance:** Data support the “ring cycle” model for dilation and constriction of the nuclear pore channel.

## Introduction

The ∼30 distinct nucleoporins (nups)^5^ building the nuclear pore complex (NPC) are organized into a gigantic protein ensemble estimated to be 120 MDa in mass in vertebrates (for review, see Ref. 1). However, an accurate copy number per NPC for each of the ∼30 nups (2, 3) has been difficult to determine experimentally as most procedures employ cell fractionation, where differential losses of nups, especially weakly attached ones, are likely to occur at the numerous theoretical plates of cell fractionation. Nevertheless, symmetry considerations provide important clues. Exhibiting an 8-fold rotational symmetry, each NPC contains at least eight (or multiples thereof) copies of a given nucleoporin. Moreover, as the central core of the NPC displays an additional 2-fold axis of symmetry (coincident with the NPC's mid-plane), the copy number for nucleoporins of the NPC core is at least 16 (or multiples thereof).

A case in point for discrepancies in reported copy numbers and stoichiometries are the three “channel” nucleoporins, Nup62, Nup54, and Nup58 (Refs. 2 and 4–8; see also Ref. 9). Each of these three nups contains an ordered region of ∼150–200 residues in length (8, 10). However, this region is not contiguous, but is subdivided into 3–4 segments of ∼40–80 residues in length, each predicted to exhibit α-helical secondary structure. Remarkably, the topology of segmentation is evolutionarily highly conserved between channel nups of uni- and multicellular eukaryotes indicating functional relevance for the segmentation of each ordered region. Notably, our mapping and crystal studies on the three channel nups matched the boundaries of these segments (9, 11, 16). Collectively, these studies led us to propose a model for the dynamics and structure of the central channel of the NPC. The salient features of this model are mid-plane rings (consisting of “dilated” and “constricted” conformers of Nup54 and Nup58) with attached triple helices of Nup62 and Nup54 (“fingers”), projecting alternately to either the nucleoplasmic or the cytoplasmic side of the mid-plane rings (9, 11).

Our structure-informed data suggested a copy number for Nup62, Nup54, and Nup58 of 128, 64, and 32 molecules, respectively, that is, a 4:2:1 stoichiometry. However, a recent study by Ulrich *et al.* (7) concluded that the three channel nups associate in a 1:1:1 stoichiometry. Ulrich *et al.* (7) arrived at this conclusion based on analytical ultracentrifugation studies of recombinant fragments comprising the entire ordered region of each of the three channel nucleoporins. However, as these authors utilized only a single loading concentration instead of the multiple loading concentrations that are required for global modeling of multi-component systems to accurately determine equilibrium constants (12–15), their conclusions are flawed *a priori*. Despite these drawbacks, Ulrich *et al.* (7) went on to suggest that the biochemically and crystal structure-defined interactomes of segments of these ordered regions (9, 11, 16) are “non-canonical” in nature and therefore of doubtful physiological relevance.

In this study, we report data on assembly and disassembly of complexes formed by the ordered region of each of the three channel nups. By employing several biochemical and biophysical approaches, we found that these fragments form dynamic assemblies of heterogeneous stoichiometries rather than forming complex(es) of a uniform 1:1:1 stoichiometry as proposed by Ulrich *et al.* (7). Importantly, we detected the previously characterized interacting domains of ordered segments of channel nups (9, 11, 16), which we refer to as interactomes, to be the principal contributors, even in the context of the heterogeneous and dynamic assemblies formed in solution by the entire ordered regions of the three channel nups. Hence, the previously detected interactomes (9, 11, 16) between segments of the ordered regions of the three channel nucleoporins are indeed “canonical.” The data reported here also provide further support for our “ring cycle” model for the molecular structure and dynamics of the central channel of the nuclear pore complex (9, 11).

## Experimental Procedures

### 

#### 

##### Plasmid Preparation, Protein Expression, and Purification

The expression construct for the Nup62(322–525)·Nup54(346–510)·Nup58(239–415) complex from *Rattus norvegicus* was generated using a polycistronic vector as described by Melčák *et al.* (16). Briefly, coding sequences of Nup58, Nup54, and Nup62 were cloned in a modular tricistronic vector with a thrombin-cleavable His_6_ tag at the N terminus of Nup58. The coding sequences of the three genes were separated by an ∼60-bp ribosome-binding site-encoding linker. Protein complex purification was performed as reported earlier (16), using His_6_ affinity chromatography (through His-tagged Nup58), followed by thrombin cleavage, anion exchange chromatography, and size-exclusion chromatography. After thrombin cleavage, a peptide sequence (GSHM) from the vector precedes the actual Nup58 sequence. Nup54 mutant H469F/Q473L was also expressed as His_6_-tagged protein from the pET28a vector and purified as described above. All the experiments were performed at 4 °C unless otherwise specified. Protein concentrations were determined from absorbance at 280 nm using extinction coefficients calculated from the protein sequence with the ProtParam tool from the ExPASy webserver (17). Secondary structure prediction was performed using PSIPRED (18).

##### Analytical Size-exclusion Chromatography

For size-exclusion chromatography analysis, 200 μl of protein sample at the given protein concentration was loaded on a Superdex200 10/300 GL column (GE Healthcare) that was pre-equilibrated with 10 mm Tris-Cl (pH 8.0), 150 mm NaCl, 1 mm DTT (buffer A), unless otherwise indicated. The column was calibrated in the same buffer with a gel filtration calibration kit (GE Healthcare) using standards of known molecular weights.

##### Circular Dichroism Spectroscopy Analysis

Secondary structure content of the complex was estimated by far UV wavelength CD scans. CD data are reported as molar ellipticities [Θ]. α-Helical content of the protein complex was estimated from the CD wavelength scans using the webserver K2D3 (19).

##### Multiangle Light Scattering

Purified proteins were subjected to analytical size-exclusion chromatography at 4 °C. The size-exclusion chromatography column (Superdex200 10/300 GL) was connected to multiangle light scattering (MALS) and refractive index detectors (DAWN HELEOS and Optilab rEX, Wyatt Technology). The temperature of the Wyatt detectors was controlled at 4 °C (the entire system was placed in a refrigeration unit kept at 4 °C). Weight-averaged molar masses were determined by MALS as described by Solmaz *et al.* (9) and averaged from at least two experiments (standard deviations were calculated; representative experiments are shown), using the ASTRA 6.0.2.9 software (Wyatt Technology).

##### Discontinuous Native Gel Electrophoresis

The analysis of proteins using native-PAGE was performed in Tris-glycine buffer (pH 8.0) according to the method of Laemmli with minor modifications. Polyacrylamide gels with 10% acrylamide in the separating gel and 4% acrylamide in the stacking gel (acrylamide:bis-acrylamide, 29:1, respectively) were used. The protein samples were mixed with equal volumes of 2× native loading buffer (250 mm Tris-Cl, pH 6.8, 0.1% bromphenol blue, and 35% glycerol). To prevent denaturation of the protein complex, the samples were run at a constant voltage of 20 V at 4 °C for 14 h. The native gel was negatively stained using the GelCode E-zinc reversible staining kit (Pierce). The identified protein bands were cut, washed with deionized water, and dried. Dry gel was crushed by a pipette tip, and the proteins were eluted with 300 μl of buffer containing 250 mm Tris-Cl (pH 6.8) and 0.1% SDS overnight at room temperature. Eluted proteins were concentrated with a 3-kDa cutoff filtration unit (Millipore). Concentrated proteins were analyzed by SDS-PAGE and silver staining (SilverXpress, Life Technologies) of the SDS-PAGE gel.

##### Matrix-assisted Depletion Assay

The MBP-Nup62(322–525)·Nup54(346–510)·Nup58(239–415) complex from *R. norvegicus* was generated by co-expression of the proteins using a polycistronic vector where N termini of Nup58 and Nup62 were tagged to His_6_ and maltose-binding protein (MBP) tags, respectively. The MBP-Nup62·Nup54·Nup58 complex was purified through His_6_ affinity chromatography. The His_6_ tag of Nup58 was cleaved with thrombin. The sample was dialyzed in buffer A. The purified protein complex was bound to amylose resin (New England Biolabs Inc.) equilibrated with buffer A. The MBP-Nup62·Nup54·Nup58-amylose resin was washed with buffer A and used for subsequent studies. 75 μl of amylose-MBP-Nup62·Nup54·Nup58 resin was incubated in a 0.8-ml centrifuge column (Thermo Scientific), at 4 °C with increasing amounts of wild-type Nup54 (453–494) in a final volume of 375 μl. The mixture was then spun at 1000 × *g* for 2 min at 4 °C. The amount of Nup58 released from the amylose matrix into the supernatant, as a function of the concentration of wild-type Nup54 (453–494), was calculated through absorbance of the supernatant at 280 nm, after the blank subtraction.

##### Limited Proteolysis and Mass Spectrometry

1 mg/ml of trypsin, chymotrypsin, and elastase stock solutions were separately added to 3 mg/ml of the Nup62·Nup54·Nup58 complex in 1:100 (v/v) ratio. Reaction mixtures were incubated at room temperature, and samples were taken out at different time points. Reactions were quenched by adding guanidine hydrochloride to a final concentration of 5 m. The protein fragments generated in the limited proteolysis experiments were identified as intact peptides measured to within 1 atomic mass unit of mass accuracy, by electrospray ionization-ion trap mass spectrometry (Bruker Amazon), after separation by microbore reversed-phase chromatography on a 300 Å pore polystyrene-divinylbenzene column (PLRP-S, Agilent).

## Results

### 

#### 

##### Modular Segmentation of Channel Nucleoporins

**FIGURE 1.**
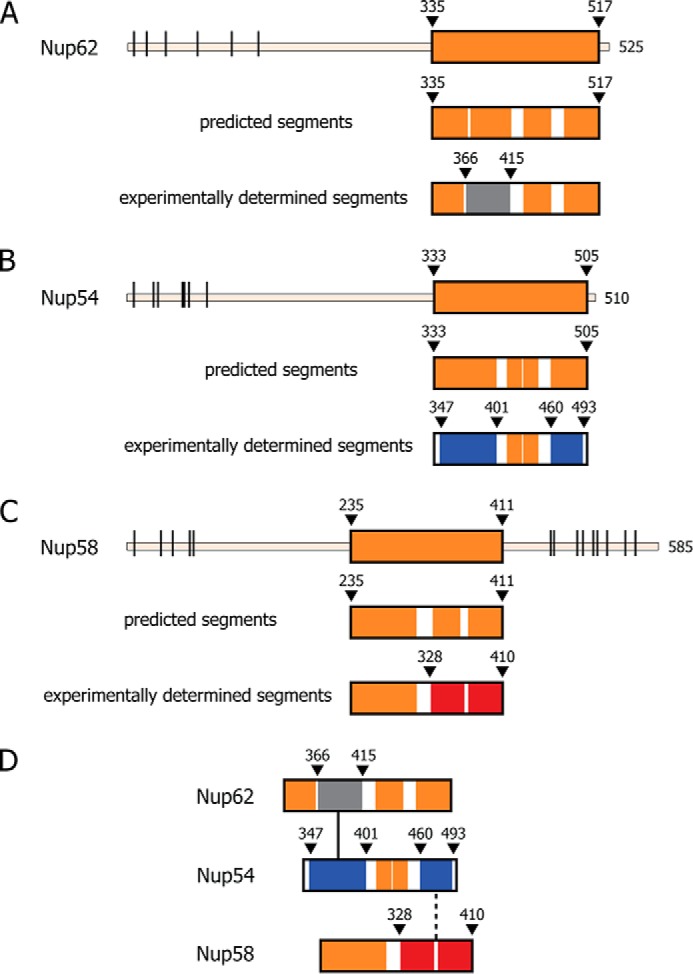
**Evolutionarily conserved segmentation of ordered regions of channel nucleoporins, Nup62, Nup54, and Nup58.** *A–C*, *top panels*, full-length Nup62, Nup54, and Nup58 from rat (*R. norvegicus*) with boundaries of each predicted ordered region *(orange-colored box*), disordered region (*thin line*), and the location of FG repeats (*black bars*) indicated. *Middle panels*, predicted α-helical segments (*orange*) and connecting loops (*white*). *Bottom panels*, ordered segments from previously determined crystal structures, color-coded as follows: *gray* for Nup62, *blue* for Nup54, and *red* for the two adjacent segments of Nup58 involved in forming a helix-loop-helix hairpin (11, 16). *D*, color-coded segments of channel nups that crystallized as hetero-interactomes (Nup54·Nup62) (*black line*) (9), and as both homo-interactomes and hetero-interactomes (Nup54·Nup58, Nup54 homo-tetramer and Nup58 homo-tetramer) (9, 11, 16). For relevant primary structures of *R. norvegicus* and *Saccharomyces cerevisiae* nups, see Fig. 2.

**FIGURE 2.**
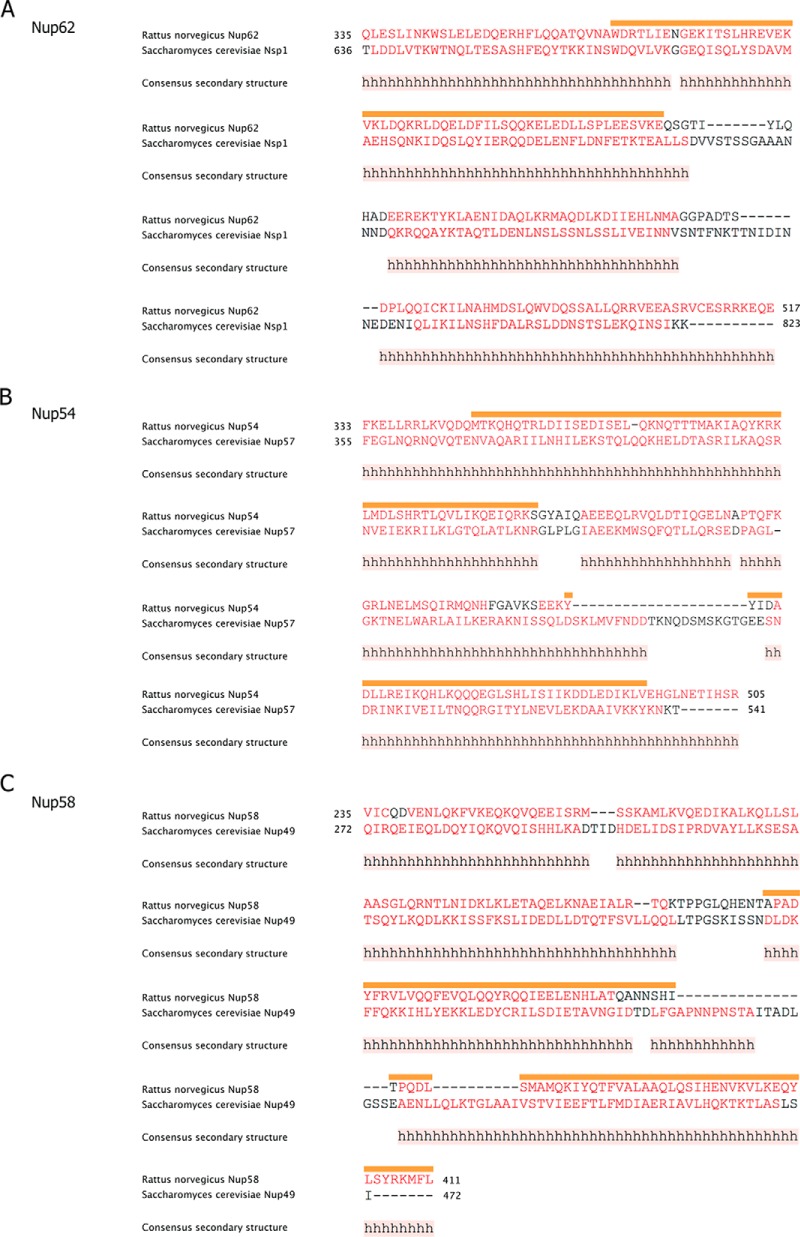
**Segmentation topology of each ordered region of the three channel nucleoporins is evolutionarily conserved.** *A–C*, sequence and predicted secondary structure comparisons of ordered region of Nup62/Nsp1 (*A*); Nup54/Nup57 (*B*); and Nup58/Nup49 (*C*) of *R. norvegicus/S. cerevisiae*, respectively, are shown. Consensus helical regions, as predicted by PROMALS3D, are labeled with *h* below the sequence alignment. Starting and ending residues of the α-helical regions used in the alignments are numbered. *Orange bar* highlights the residues of the regions for which x-ray structures are known from *R. norvegicus* (9, 11, 16).

The channel nups Nup62, Nup54, and Nup58 contain ordered regions of ∼150–200 residues and natively unfolded regions of up to ∼300 residues that are marked by repeating phenylalanine-glycine (FG) motifs. Fig. 1 (*A–C*) illustrates the boundaries of the predicted ordered region in each of the three channel nups and indicates segmentation of each region into 3–4 predicted α-helical segments of ∼40–80 amino acid residues in length. As indicated (Fig. 1*D*), the boundaries of the predicted helical segments of each ordered region closely matched the experimental data (9, 11, 16) we obtained from mapping and crystal structure studies (Fig. 1, *A–D*). This segmentation pattern is highly conserved between uni- and multicellular eukaryotes (Fig. 2). Each of these segments has been traced and assigned to modular structures that build mid-plane rings and their projecting fingers in a model of a reconstructed central channel, in both its dilated and its constricted forms (11) (Fig. 1*D*).

##### Biophysical and Biochemical Analyses of Triple Complex

**FIGURE 3.**
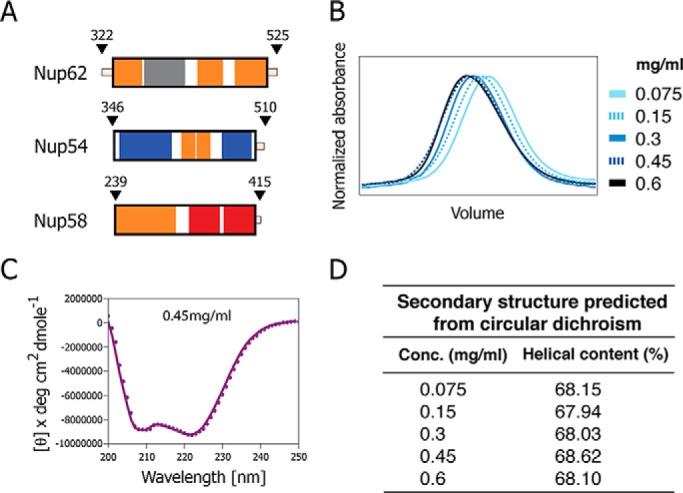
**Size-exclusion chromatography elution profiles and circular dichroism of triple complex assembled from the ordered regions of Nup62, Nup54, and Nup58.** *A*, boundaries for each ordered region of channel nups used for the triple complex formation color-coded as Fig. 1*D. B*, elution profiles at increasing concentrations of the triple complex in size-exclusion chromatography, monitored by the absorbance at 280 nm. *C*, circular dichroism wavelength scan of molar ellipticity of the triple complex acquired at 0.45 mg/ml. Negative ellipticity minima at 208 and 222 nm indicate that the triple complex is primarily α-helical. *deg*, degrees. *D*, α-helical contents of triple complexes at increasing concentrations (*Conc.*) determined by CD spectroscopy.

We purified a “triple complex” comprising the entire ordered region of each of the three channel nups (Fig. 3*A*). Size-exclusion chromatography of the purified triple complex at increasing concentrations showed a corresponding shift of its broadly eluting peak to earlier elution positions (Fig. 3*B*) (16), consistent with dynamic and concentration-dependent mass heterogeneity of the triple complex. We analyzed the complex by circular dichroism spectroscopy to determine its secondary structure content. Wavelength spectra of the triple complex showed no significant difference at various concentrations (0.075–0.6 mg/ml) on the α-helical content of the structure, which was calculated to be about 68% (Fig. 3, *C* and *D*). This value closely matched the predicted α-helical content for the complex (Fig. 2). Hence, the observed concentration effects on the elution profile (Fig. 3*B*) (16) are not attributable to improper folding of α-helical regions.

**FIGURE 4.**
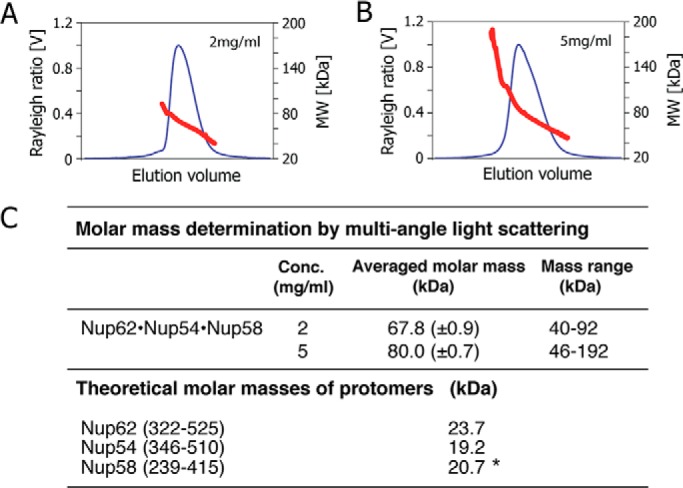
**Analysis of triple complex by size-exclusion chromatography coupled to MALS.** *A* and *B*, Rayleigh ratio (*blue line*) and molar masses (*red line*) were determined at two concentrations (*A*, 2 mg/ml; *B*, 5 mg/ml) across the elution peak. *MW*, molar mass. *C*, summary of averaged molar masses and of mass ranges for the triple complex at two concentrations (*Conc.*), and theoretical molar masses of the three nucleoporins; *asterisk* denotes the mass including the peptide sequence (GSHM) from the vector that precedes the actual Nup58 sequence.

To assess the range of mass heterogeneity of the broadly eluting peak of the triple complex, we coupled size-exclusion chromatography to MALS analyses to determine molar masses (Fig. 4, *A* and *B*). The large range in molar masses exhibited by the entirety of the eluted peak is listed in Fig. 4*C*. Notably, the molar mass distribution across the peak ranges from 40 to 92 kDa at 2 mg/ml and from 46 to 192 kDa at 5 mg/ml. Within the considerable mass heterogeneity across the elution profile of the triple complex, masses as low as 40–46 kDa (Fig. 4*C*) were detected in the descending part of the peak. As the theoretical mass of a 1:1:1 triple complex would be ∼64 kDa, the descending part of the peak is made up of two rather than three protein molecules. It should also be noted that in a mixture of heterogeneous assemblies that are in continuous dynamic equilibrium, the individual oligomeric states are not fully separated, and therefore their masses are averaged. Thus, individual assemblies with lower or higher mass may exist. The mass distribution of the ternary complex and its concentration-dependent variability support the observation that the triple complex is heterogeneous and forms multiple oligomeric states. Furthermore, the mass distribution accommodates various structural states expected from our model of the NPC transport channel, and it can be excluded that the complex forms a stable and homogenous 1:1:1 assembly, as suggested previously (7).

**FIGURE 5.**
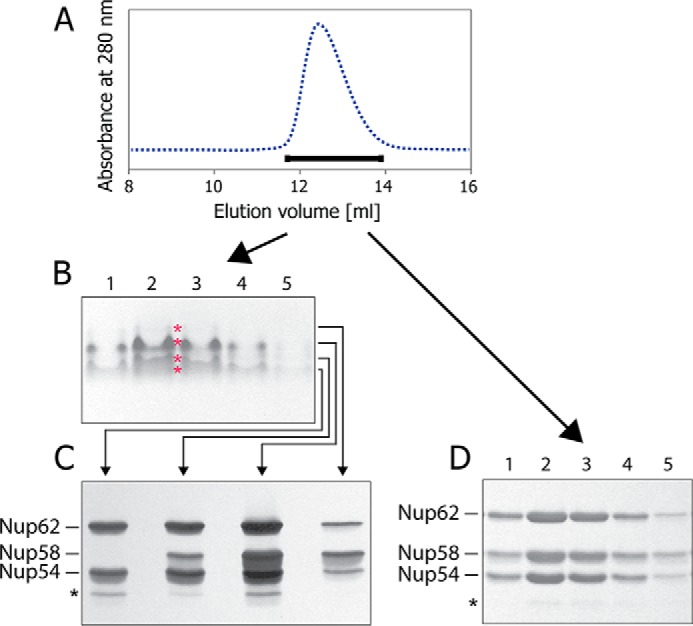
**Heterogeneity of triple complex detected by multidimensional analysis.** *A*, fractions across the size-exclusion chromatography peak (*horizontal bar*) were used for electrophoresis. *B*, native PAGE. “Iso-electrophoretic” bands were cut separately (marked by *red asterisks*). *C*, proteins from these different bands were separated by SDS-PAGE. *D*, fractions across the size-exclusion chromatography peak (*A*) analyzed by SDS-PAGE directly.

To further assess the degree of heterogeneity of the triple complex, we used multidimensional analysis. Aliquots from fractions across the peak eluted from size-exclusion chromatography (Fig. 5*A*) were first subjected to electrophoresis under native conditions (Fig. 5*B*), followed by electrophoresis of the resulting resolved fractions under denaturing conditions (SDS) (Fig. 5*C*). We observed that each of the four analyzed fractions was composed of distinct mixtures of varying composition of the three channel nups. Notably, the amount of Nup58 is variable. For example, one of these four fractions (Fig. 5*B*) contained primarily Nup62 and Nup54 when analyzed in a second dimension under denaturing conditions (Fig. 5*C*). When only one-dimensional SDS-PAGE analysis was carried out on fractions across the peak eluted by size-exclusion chromatography (Fig. 5*A*), the staining intensity for the three components of the triple complex appeared to be consistent with 1:1:1 stoichiometry of Nup62, Nup54, and Nup58 (Fig. 5*D*), thus camouflaging the dynamic heterogeneity of the triple complex as illustrated in Fig. 5, *B* and *C*.

##### Disrupting Interactions in the Triple Complex

**FIGURE 6.**
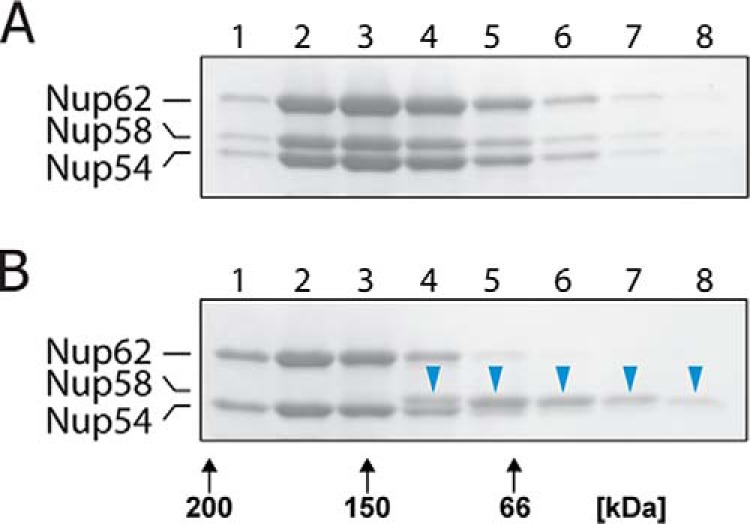
**The triple complex is partially disassembled by non-ionic detergent.** *A* and *B*, the triple complex (5 mg/ml) was subjected to size-exclusion chromatography in the absence (*A*) or presence of 65 mm *n*-octyl-β-d-glucopyranoside (*B*) followed by SDS-PAGE of column fractions. *Blue arrowheads* in *B* indicate that Nup58 was dissociated from the triple complex; *black arrows* indicate elution positions of molecular weight markers.

To probe the stability of the triple complex, we subjected it to size-exclusion chromatography in the presence of the non-ionic detergent *n*-octyl-β-d-glucopyranoside. Strikingly, the Nup58 component of the triple complex was dissociated during size-exclusion chromatography in 65 mm *n*-octyl-β-d-glucopyranoside and eluted in later fractions, separated from the peak of complexes containing Nup54 and Nup62 (Fig. 6). These data indicate that Nup58 engages in triple complex formation primarily through hydrophobic interactions, in agreement with our crystal data showing the Nup54·Nup58 interface to be largely hydrophobic in nature (9).

**FIGURE 7.**
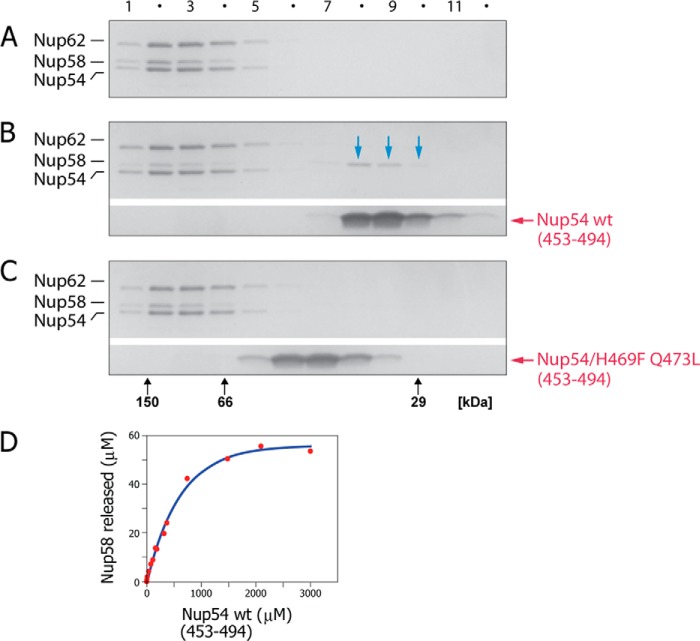
**The Nup58-cognate segment of Nup54 is solely responsible for tying the ordered region of Nup58 to the triple complex.** *A–C*, triple complex (1.5 mg/ml) was incubated in the absence (*A*) or in the presence of Nup54 (453–494), using either wild type (0.6 mg/ml) (*B*) or mutant H469F/Q473L (0.6 mg/ml) (*C*), followed by size-exclusion chromatography of the incubated sample and subsequent SDS-PAGE of column fractions. Note, that Nup58 (*blue arrows* in *B*) is partially removed from the triple complex by wt Nup54 (453–494) (*B*), but not by mutant Nup54. *D*, in a matrix depletion assay, where triple complex is immobilized via a Nup62-attached MBP tag, increasing concentrations of wt Nup54 (453–494) led to increasing Nup58 removal.

In a second approach, we probed the preassembled triple complex in a competition assay with a cognate segment of Nup54 (residues 453–494) as used for determination of the crystal structure of the Nup54·Nup58 interactome (9). These components were incubated, and the mixture was subjected to size-exclusion chromatography followed by SDS-PAGE analysis (Fig. 7). Wild-type Nup54 segment pulled Nup58 (comprising the entire ordered region of Nup58) out of the triple complex (Fig. 7*B*). Competition was quantitated using a matrix-assisted depletion assay (20) in which amylose resin-bound MBP triple complex was titrated with varying amounts of wild-type Nup54 (453–494). The amount of Nup58 depleted from the matrix was monitored through increase in the absorbance in the solution. Indeed, incubation of the triple complex with increasing concentrations of Nup54 segment pulled out increasing amounts of Nup58 (Fig. 7*D*). We conclude that Nup58 is anchored to the triple complex primarily via its interaction with a cognate segment of Nup54 (9) and that interactions of other segments of Nup58, if they would contribute to triple complex formation, would be too weak to detect in this assay. Strikingly, a thermostable mutant of the Nup54 segment (11) where two crucially localized hydrophilic residues were changed to hydrophobic ones (H469F and Q473L) was unable to accomplish an equivalent pullout of Nup58 from the triple complexes (Fig. 7*C*). Together these data are consistent with the previously identified Nup54·Nup58 interactome (9) being an indispensable contributor for triple complex formation.

**FIGURE 8.**
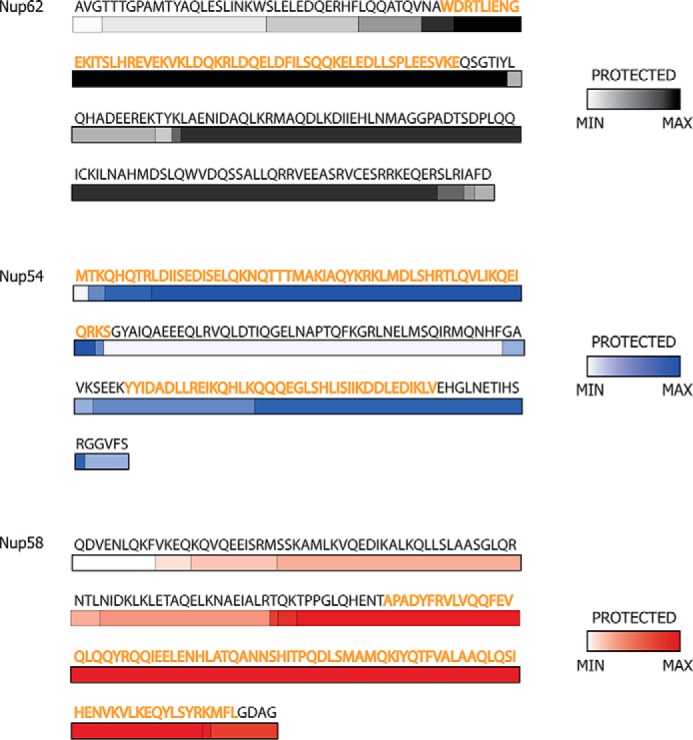
**Protection from limited proteolysis of the triple complex in solution matches regions of cognate segments of previously determined structures.** The color gradient of each bar represents the frequency of a fragment observed in mass spectrometry after limited proteolysis: *white* represents the least protected region, whereas the most protected regions are depicted by *dark gray* (Nup62), *dark blue* (Nup54), and *dark red* (Nup58). Residues of the regions for which x-ray structures are known are colored as *orange*.

In a third approach, we exposed the triple complex to various proteases and identified the resulting fragments by mass spectrometry, with the expectation that interacting regions should be more protected from proteolysis. Strikingly, the location of protease-protected regions of the triple complex roughly coincided with the boundaries of our previously identified and characterized interactomes (9, 11, 16), which form the principal elements (mid-plane rings and fingers) in our model of a reconstructed channel of the nuclear pore (Figs. 1*D* and 8).

##### Cognate Segments Are Sufficient for Triple Complex Assembly

**FIGURE 9.**
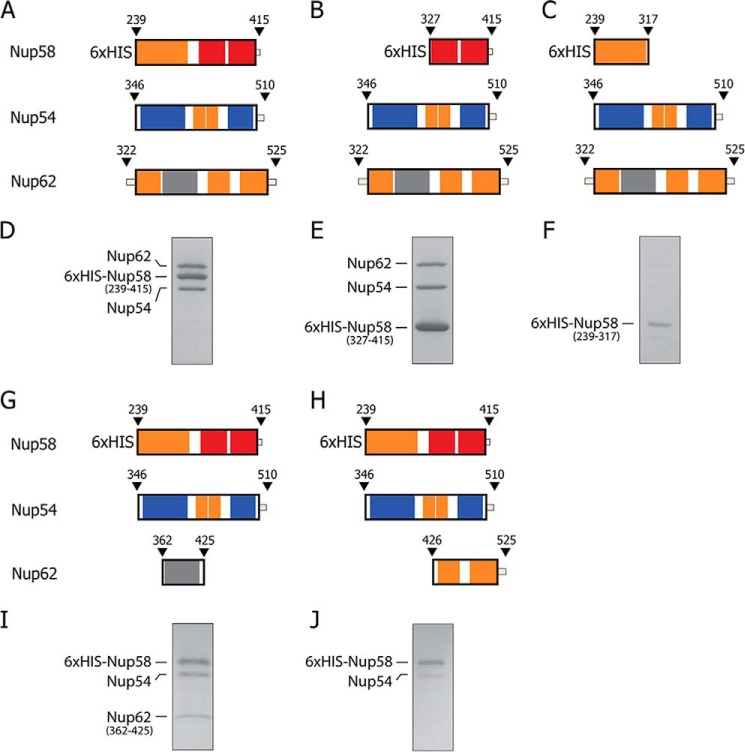
**Formation of triple complex requires cognate segments of Nup58 and Nup62.** His_6_-tagged Nup58, representing its full-length ordered region (*A*) or its C-terminal segment (*B*) (cognate for Nup54), pull down a triple complex, when co-expressed with Nup54 and Nup62, as assessed by SDS-PAGE (*D* and *E*, respectively). In contrast, His_6_-tagged Nup58 containing only the N-terminal segment of the ordered region of Nup58 is unable to efficiently pull down a triple complex (*C* and *F*). Using His_6_-tagged Nup58 (representing its ordered region) for affinity pulldown and testing truncated forms of the ordered region of Nup62, only the N-terminal segment of Nup62 (cognate to the N-terminal segment of Nup54) (*G*) was incorporated into a triple complex (*I*), whereas the C-terminal segment of Nup62 (*H*) was not (*J*).

To test whether the previously defined interaction domains between the cognate segments (9, 11, 16), either of Nup54 and Nup58 or of Nup54 and Nup62, are indeed principal contributors to the triple complex assembly, we co-expressed various deletion constructs of the three channel nups, purified the resulting complexes by affinity pulldown via the His_6_ tag of Nup58, and analyzed the eluates by SDS-PAGE (Fig. 9). We found an N-terminally truncated region of Nup58 (Fig. 9, *B* and *E*), but not its C-terminally truncated counterpart (Fig. 9, *C* and *F*), to be sufficient to efficiently co-purify Nup62 and Nup54 (compare with Fig. 9, *A* and *D*). Moreover, a truncated Nup54 cognate segment of Nup62, but not an N-terminally truncated counterpart of Nup62, could be co-purified with Nup54 and Nup58 (Fig. 9, *G–J*). These data confirm that our crystallographically defined Nup54·Nup58 and Nup54·Nup62 hetero-interactomes are indeed crucial for forming the heterogeneous and dynamic assemblies of the triple complex.

## Discussion

Here, we report solution studies on a triple complex formed by the ∼150–200-residue-long ordered region of each of the three channel nucleoporins, Nup62, Nup54, and Nup58. We show that recombinant proteins (each representing the entire ordered region of the three channel nups) form a dynamic mixture of heterogeneous complexes. We biophysically and biochemically characterized this triple complex and employed truncated forms or cognate segments of the ordered region for probing assembly and disassembly of the triple complex. We find that our previously identified hetero-interactomes (9) formed by cognate segments of the ordered region of the three channel nups are indeed also the principal drivers for triple complex assembly in solution.

**FIGURE 10.**
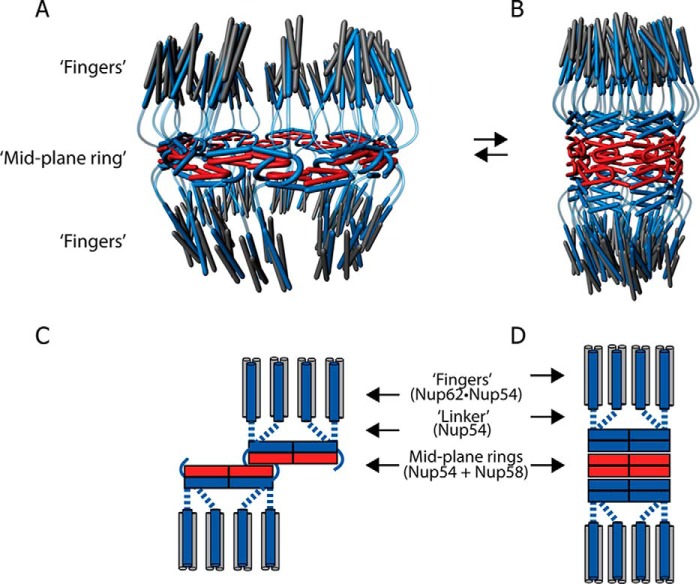
**Piecing together crystal structures of channel nucleoporin segments into a model for a nuclear pore channel.** *A* and *B*, schematic representation of inter-convertible dilated (*A*) and constricted (*B*) channel states. The path of segments of ordered regions of channel nups, Nup62 (*gray*), Nup54 (*blue*), and Nup58 (*red*), through the channel's two principal structural elements, mid-plane ring and attached fingers, is shown. The latter are projecting to nucleoplasm and cytoplasm. *C* and *D*, a single module (out of eight) is represented each for the dilated (*C*) or constricted (*D*) states of the channel. The “linker region” of Nup54 is indicated either by *curved lines* (*A* and *B*) or by *dotted lines* (*C* and *D*).

Cognate segments of the ordered region of each of the channel nups have previously been identified and characterized by mapping and atomic resolution crystal structures, which led us to propose a model for the structure and dynamics of the central channel of the nuclear pore (9, 11). Two salient structural features of this model are mid-plane rings and attached triple helices (fingers) that form cytoplasmic and nucleoplasmic entries to the mid-plane rings (Fig. 10). With regard to dynamics, we have proposed a ring cycle model, whereby conformers of a *single large* (dilated) ring convert to conformers of *three small* (constricted) rings, reflecting opened and closed states of the central channel of the nuclear pore (Fig. 10), with transport factors suggested to stabilize the open form of the central channel (9, 11). Indeed, a recent study yielded data in support of the proposed regulation of the central transport channel. Transport factor binding to the Nup58 segment (extended C-terminally to contain the requisite phenylalanine-glycine repeat binding site for the transport factor) was shown to *allosterically* stabilize dilated Nup54·Nup58 conformers, at the expense of their constricted counterparts (21).

Collectively, our studies led to crystal structure-informed copy numbers of 128, 64, and 32 molecules of Nup62, Nup54, and Nup58, respectively, amounting to a mass of 12.3 MDa for the central channel, roughly 10% of the estimated mass of the entire NPC (9). Our model also suggests that the hitherto unassigned segments of the ordered regions of the three channel nups provide binding sites for neighboring nups. Specifically, a segment located in the N-terminal part of the ordered region of Nup58 was proposed to anchor mid-plane rings to neighboring nucleoporins, and a C-terminal segment of the ordered region of Nup62 was proposed to link the base of the fingers to a neighboring nucleoporin. Although structural information for interactions of channel nups with neighboring nups is still missing, there is biochemical evidence for this arrangement (22, 23). Hence, the path of segments of the ordered regions of each of the three channel nucleoporins has been tentatively traced (11) and assigned to both the principal elements of the central channel (mid-plane ring and fingers) and putative binding sites in neighboring nucleoporins. Of the three channel nups, Nup54 stands out as it provides two cognate segments, one for forming the mid-plane ring and the other for forming the fingers, respectively, whereas only one segment each of Nup62 and two adjacent segments of Nup58 (Fig. 1*D*) contribute to the finger and mid-plane elements of the central channel. Notably, the structure-informed stoichiometry of 4:2:1 for Nup62, Nup54, and Nup58 has been supported by *in vivo* quantitative fluorescence intensity data from the fission yeast where endogenous nups were genomically replaced with their GFP-tagged versions (24).

Our present solution studies on the triple complex are in support of our proposals for tracing the segments of the ordered region (Fig. 10) (11). We show that the principal interactions of the triple complex are established by the Nup54·Nup58 interactome (responsible for mid-plane ring) and the Nup54·Nup62 interactome (building the fingers) (Figs. 9 and 10). Accordingly, the hitherto “unsaturated” segments of Nup58 and Nup62 that potentially link the structural elements of the central channel to neighboring nucleoporins do not participate in assembly or in disassembly of the triple complex.

Collectively, these studies on the triple complex are entirely consistent with our previous mapping and crystal studies (9, 11, 16) and validate our approach not only to gain insight into the dynamics and structure of the central channel of the NPC, but also to probe the structure and dynamics of surrounding nucleoporins, which are likely to undergo structural and dynamic changes accompanying and accommodating the huge diameter changes proposed for the central channel of the NPC (9, 11).

## Author Contributions

A. S, S. R. S., G. B., and I. M. designed experiments, analyzed data, and wrote the paper. A. S., S. R. S., and I. M. performed experiments.

## Footnotes

5 The abbreviations used are:

nup: nucleoporin
NPC: nuclear pore complex
MALS: multiangle light scattering
MBP: maltose-binding protein.
